# Operational Infection Control Strategies for Clinic Staff During the Pandemic: A Multi-Facility Questionnaire Study in Japan

**DOI:** 10.31662/jmaj.2026-0020

**Published:** 2026-04-24

**Authors:** Kei Ijichi, Hiroshi Yotsuyanagi

**Affiliations:** 1 Akaike ENT-HNS Clinic, Nissin, Japan; 2 Division of Infectious Diseases, Advanced Clinical Research Center, The Institute of Medical Science, The University of Tokyo, Tokyo, Japan

**Keywords:** COVID-19, infection control, outpatient clinics, healthcare workers, pandemic preparedness

## Abstract

**Background:**

During the coronavirus disease 2019 (COVID-19) pandemic, outpatient clinics were required to continue providing routine medical care amid uncertainty, limited resources, and concerns about staff safety. However, evidence describing operational infection control measures in clinic settings remains limited.

**Methods:**

We conducted a cross-sectional questionnaire survey of medical facilities affiliated with the Minato Ward Medical Association in Tokyo and the Higashi Nagoya Medical Association in Aichi Prefecture, Japan. The survey was conducted between December 2023 and January 2024. The survey assessed facility characteristics, staffing patterns, infection control measures implemented during the COVID-19 pandemic, methods of information sharing among staff, and experiences of social impact such as discrimination. Descriptive statistics were used, and regional comparisons were performed where appropriate.

**Results:**

Responses were obtained from 63 medical facilities, primarily outpatient clinics. Common infection control measures for staff included installing acrylic panels and protective sheets at reception areas, providing personal protective equipment, regularly monitoring temperatures, and canceling in-person staff gatherings. These measures were implemented in a substantial proportion of facilities across regions. Regarding information sharing, the use of digital communication tools was more frequent in clinics located in Tokyo than in those in Aichi Prefecture. A small proportion of facilities reported experiences of discrimination against staff, with no marked regional differences observed.

**Conclusions:**

Outpatient clinics in Japan rapidly adopted practical infection control measures to protect staff during the COVID-19 pandemic. While many measures were universally implemented, certain operational practices, such as information-sharing methods, varied across regions. These findings provide practical insights into infection control preparedness for outpatient clinics facing future infectious disease emergencies.

## Introduction

The coronavirus disease 2019 (COVID-19) pandemic posed unprecedented challenges to healthcare systems worldwide, requiring medical institutions to continue providing routine care amid uncertainty and limited resources ^(1)^. Healthcare workers were disproportionately affected during the pandemic, with a high burden of occupational infection reported across multiple countries ^(2), (3)^.

Previous studies have demonstrated that a substantial proportion of infections among healthcare workers occurred outside hospital settings, highlighting the complexity of infection risk during the pandemic ^(3), (4)^. Although hospitals have been the primary focus of research on infection control measures, outpatient clinics represent a critical yet understudied setting. Clinics serve as frontline points of contact for symptomatic patients and play an essential role in maintaining access to general and primary medical care.

Unlike large hospitals, outpatient clinics often operate with limited staffing, space, and equipment, which may constrain the implementation of infection control protocols designed for hospital environments. During the early phase of the COVID-19 pandemic, clinics were required to make rapid operational decisions on staff protection, often in the absence of clear, consistent guidelines. In addition to infection risk, clinic staff faced psychological and social pressures, including fear of transmitting infection to family members and experiences of stigma or discrimination within the community ^(2), (5)^.

Although several studies have examined infection prevention measures and the experiences of healthcare workers during the COVID-19 pandemic ^(5), (6)^, evidence on how outpatient clinics operationalized infection control measures for staff remains limited. Furthermore, little is known about whether such measures were uniformly adopted across regions or varied according to local context.

The purpose of this study was to examine infection control measures implemented for clinic staff during the COVID-19 pandemic in outpatient clinic settings in Japan. Using a multi-facility questionnaire survey conducted in both urban and suburban regions, we aimed to identify infection control practices widely adopted across facilities, and operational strategies that differed by regional or institutional context. By clarifying these patterns, this study seeks to provide practical insights that may inform preparedness for future infectious disease outbreaks.

## Materials and Methods

### Study design

This study was a cross-sectional questionnaire survey conducted to assess infection control measures for clinic staff during the COVID-19 pandemic. The survey captured retrospective information on infection control practices during the COVID-19 pandemic and current practices at the time of the survey.

### Study setting and participants

The survey was conducted among medical facilities affiliated with the Minato Ward Medical Association in Tokyo and the Higashi Nagoya Medical Association in Aichi Prefecture (including Nisshin City, Nagakute City, Toyoake City, and Togo Town), Japan. The Minato Ward Medical Association in Tokyo and the Higashi Nagoya Medical Association in Aichi Prefecture were selected as study sites to represent outpatient clinic settings in urban and suburban regions of Japan, respectively. Minato Ward is a central metropolitan area of Tokyo characterized by a high daytime population and a large concentration of medical facilities, whereas the Higashi Nagoya area comprises suburban municipalities with mixed residential and commercial characteristics. These medical associations were chosen because they include a broad range of outpatient clinics providing primary and general medical care and maintain established communication networks that facilitated distribution of the questionnaire. This approach allowed efficient data collection during the post-pandemic period while minimizing the burden on participating clinics. Because participating clinics were limited to those affiliated with these medical associations, selection bias cannot be excluded, and caution is required when generalizing the findings to all outpatient clinics in Japan. Minato Ward has a residential population of approximately 260,000, with a substantially larger daytime population, while the Higashi Nagoya area includes municipalities with a combined population of approximately 430,000. Eligible participants included outpatient clinics and small hospitals that provided routine medical care during the COVID-19 pandemic. Participation was voluntary, and all responses were collected anonymously at the facility level.

### Survey content

The questionnaire was developed to assess operational infection control measures implemented for clinic staff during the COVID-19 pandemic. The survey consisted of items covering the following domains: (1) clinic characteristics, including facility type and staffing; (2) infection control measures, such as the use of physical barriers, personal protective equipment (PPE), and staff health monitoring; (3) methods of information sharing related to COVID-19, including digital communication tools and face-to-face meetings; and (4) experiences of social impacts, including discrimination against clinic staff. With regard to information sharing, digital communication tools included messaging applications, e-mail, and web-based platforms used for rapid dissemination of information among staff. Respondents were asked to select all applicable items.

The full questionnaire used in this study is available from the authors upon reasonable request.

### Data collection

The questionnaire survey was conducted between December 20, 2023, and January 31, 2024. Respondents were asked to report infection control measures implemented during the COVID-19 pandemic, including practices adopted at its peak and those maintained up to the time of the survey. The survey was distributed to affiliated medical facilities through their respective medical associations. Completed questionnaires were returned and aggregated for analysis.

### Statistical analysis

Statistical analyses were performed using R version 4.4.0 (R Foundation for Statistical Computing, Vienna, Austria) and EZR version 1.66 (Saitama Medical Center, Jichi Medical University, Japan) ^(7)^, a graphical user interface for R designed for medical statistics. Categorical variables were analyzed using Fisher’s exact test. A two-sided p-value of <0.05 was considered statistically significant.

### Ethical considerations

This study was approved by the Institutional Review Board of the Institute of Medical Science, The University of Tokyo (approval number: 2023-23-0720). The requirement for individual informed consent was waived because the survey was conducted at the facility level, responses were anonymous, and no identifiable personal information was collected.

## Results

The reported practices reflect infection control measures implemented during the COVID-19 pandemic and those in place at the time of the survey.

### Facility characteristics

A total of 63 medical facilities responded to the survey, including 37 in Tokyo and 26 in Aichi Prefecture. Table 1 summarizes the characteristics of participating medical facilities. The majority of participating facilities were outpatient clinics, with only a small number of small hospitals included. Most facilities primarily provided internal medicine services and treated <100 outpatients per day. Most operated without inpatient beds and had relatively small staffing structures, typically consisting of one to several physicians, a limited number of nurses, and a small administrative staff (Table 1).

**Table 1.** Characteristics of participating medical facilities.

Type of facility	Total (n = 63)	Tokyo (n = 37)	Aichi (n = 26)
Outpatient clinic	57 (90.5%)	33 (89.2%)	24 (92.3%)
Hospital	6 (9.5%)	4 (10.8%)	2 (7.7%)
**Main department**			
Internal medicine	31 (49.2%)	22 (59.5%)	9 (34.6%)
Otorhinolaryngology	9 (14.3%)	2 (5.4%)	7 (26.9%)
Pediatrics	4 (6.3%)	2 (5.4%)	2 (7.7%)
Orthopedics	3 (4.8%)	1 (2.7%)	2 (7.7%)
**Average number of outpatients per day**			
<50	27 (42.9%)	18 (48.7%)	9 (34.6%)
50-100	25 (39.7%)	13 (35.1%)	12 (46.2%)
>100	11 (17.5%)	6 (16.2%)	5 (19.2%)
**Inpatient beds available**			
Yes	4 (6.3%)	1 (2.7%)	3 (11.5%)
No	59 (93.7%)	36 (97.3%)	23 (88.5%)
**Number of physicians**			
1	33 (36.5%)	14 (37.8%)	19 (73.1%)
2-3	19 (30.2%)	14 (37.8%)	5 (19.2%)
≥4	11 (17.5%)	9 (24.3%)	2 (7.7%)
**Number of nurses**			
0-1	16 (25.4%)	14 (37.9%)	2 (7.7%)
2-3	25 (39.7%)	11 (29.7%)	14 (53.8%)
≥4	22 (34.9%)	12 (32.4%)	10 (38.5%)
**Number of administrative staff**			
0-1	9 (14.3%)	9 (24.3%)	0 (0.0%)
2-4	38 (60.3%)	21 (56.8%)	17 (65.4%)
≥5	16 (25.4%)	7 (18.9%)	9 (34.6%)

Data are presented as numbers (%). Percentages may not total 100 because of rounding. Tokyo includes facilities affiliated with the Minato Ward Medical Association. Aichi includes facilities affiliated with the Higashi Nagoya Medical Association (Nisshin City, Nagakute City, Toyoake City, and Togo Town). Facilities were classified according to their primary department. Hospitals are small-scale facilities that provide outpatient services.

**Figure 1.**
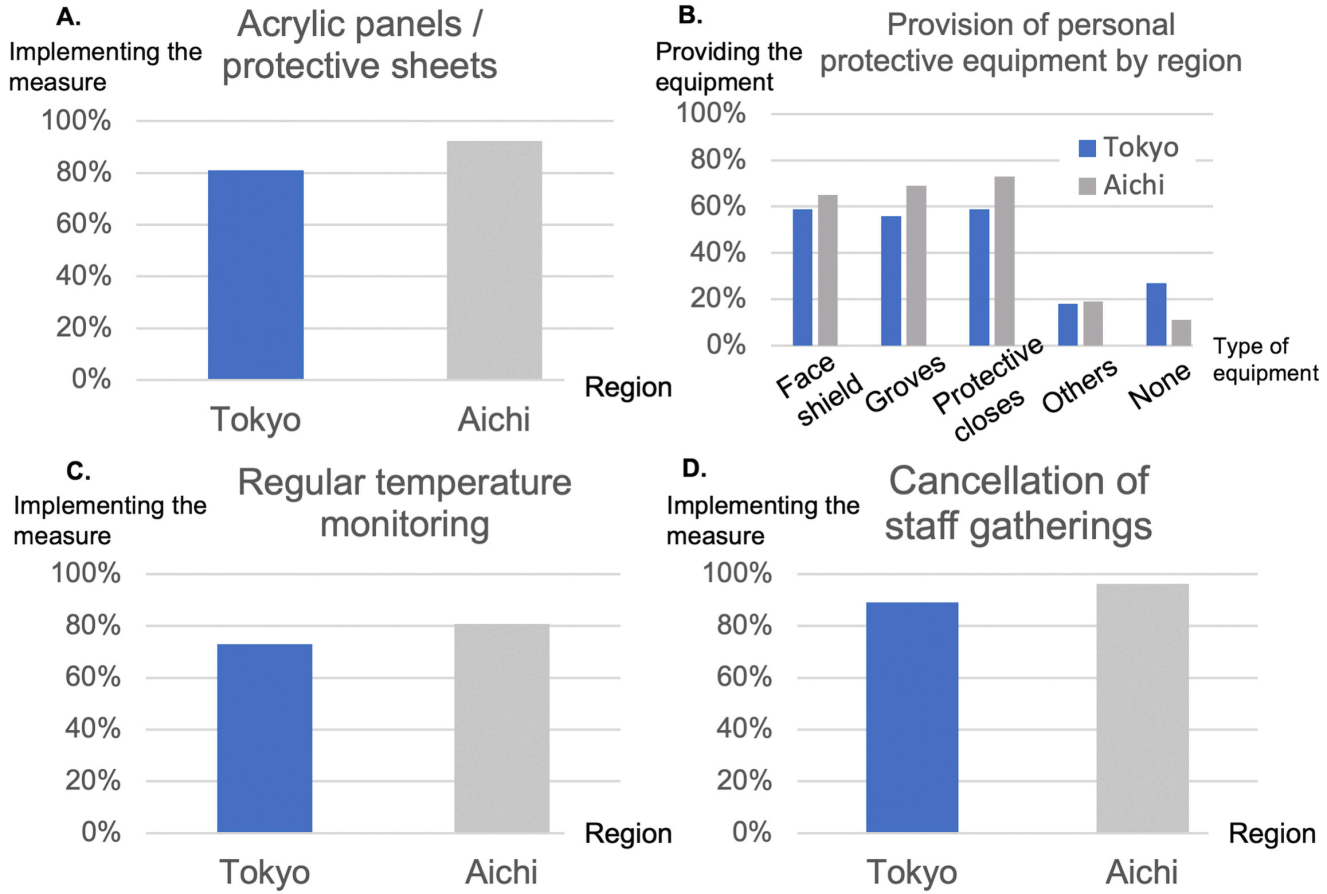
Infection control measures were implemented for clinic staff during the COVID-19 pandemic. Panels A-D show the proportion of outpatient clinics implementing each infection control measure, stratified by region (Tokyo and Aichi Prefecture). Panel A illustrates the use of physical barriers; Panel B shows the provision of personal protective equipment; Panel C depicts staff health-monitoring practices; and Panel D represents methods of information sharing among staff. Comparisons between regions were performed using Fisher’s exact test. No statistically significant regional differences were observed. COVID-19: coronavirus disease 2019.

Infection control measures for clinic staff during the COVID-19 pandemic are shown in Figure 1. Installation of acrylic panels or protective sheets at reception areas was widely implemented in both Tokyo and Aichi Prefecture (Figure 1A). Similarly, regular temperature monitoring of staff (Figure 1C) and cancellation of in-person staff gatherings, such as year-end or farewell parties (Figure 1D), were commonly reported across regions. No marked regional differences were observed for these measures.

Provision of PPE, including face shields, gloves, and protective clothing, was implemented by a substantial proportion of participating facilities (Figure 1B). When stratified by region, no statistically significant differences were observed between clinics in Tokyo and Aichi. The remaining facilities reported limited or selective use of PPE depending on availability and operational needs. Overall, these findings indicate that core infection control measures for clinic staff were implemented in a largely uniform manner across regions.

### Information sharing among staff

**Figure 2.**
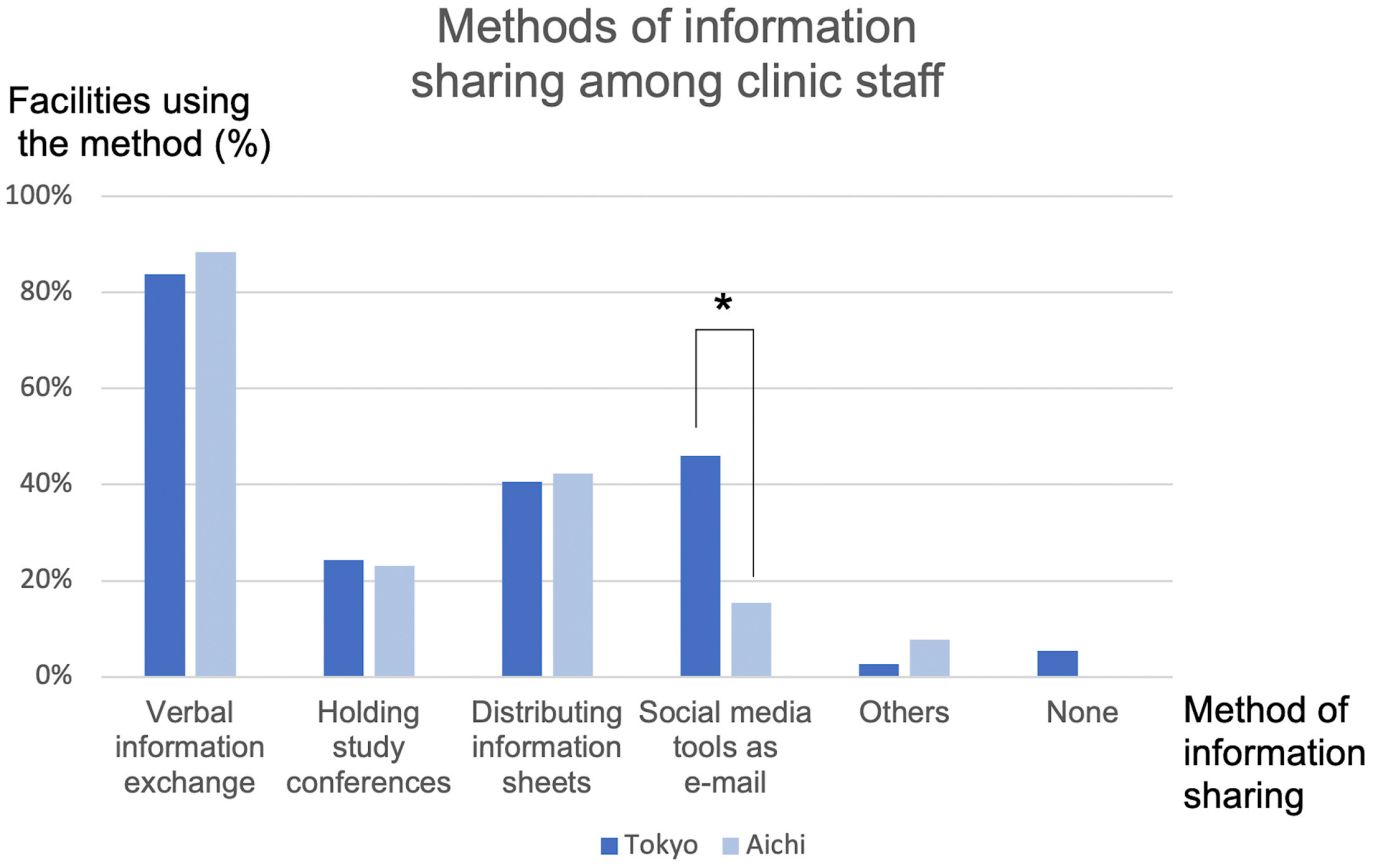
Methods of information sharing related to COVID-19 among clinic staff. Bars indicate the proportion of clinics using each method. Comparisons between regions were performed using Fisher’s exact test (*p < 0.05). COVID-19: coronavirus disease 2019.

Methods of information sharing related to COVID-19 are shown in Figure 2. Verbal communication among staff was the most commonly reported method. Distribution or display of written information was also frequently used. The use of digital communication tools, such as social media or messaging applications, was reported by approximately one-third of facilities.

When analyzed by region, facilities in Tokyo reported more frequent use of digital communication tools than those in Aichi Prefecture, a difference that was statistically significant (p = 0.0148). No marked regional differences were observed for other information-sharing methods.

### Social and operational impacts

**Figure 3.**
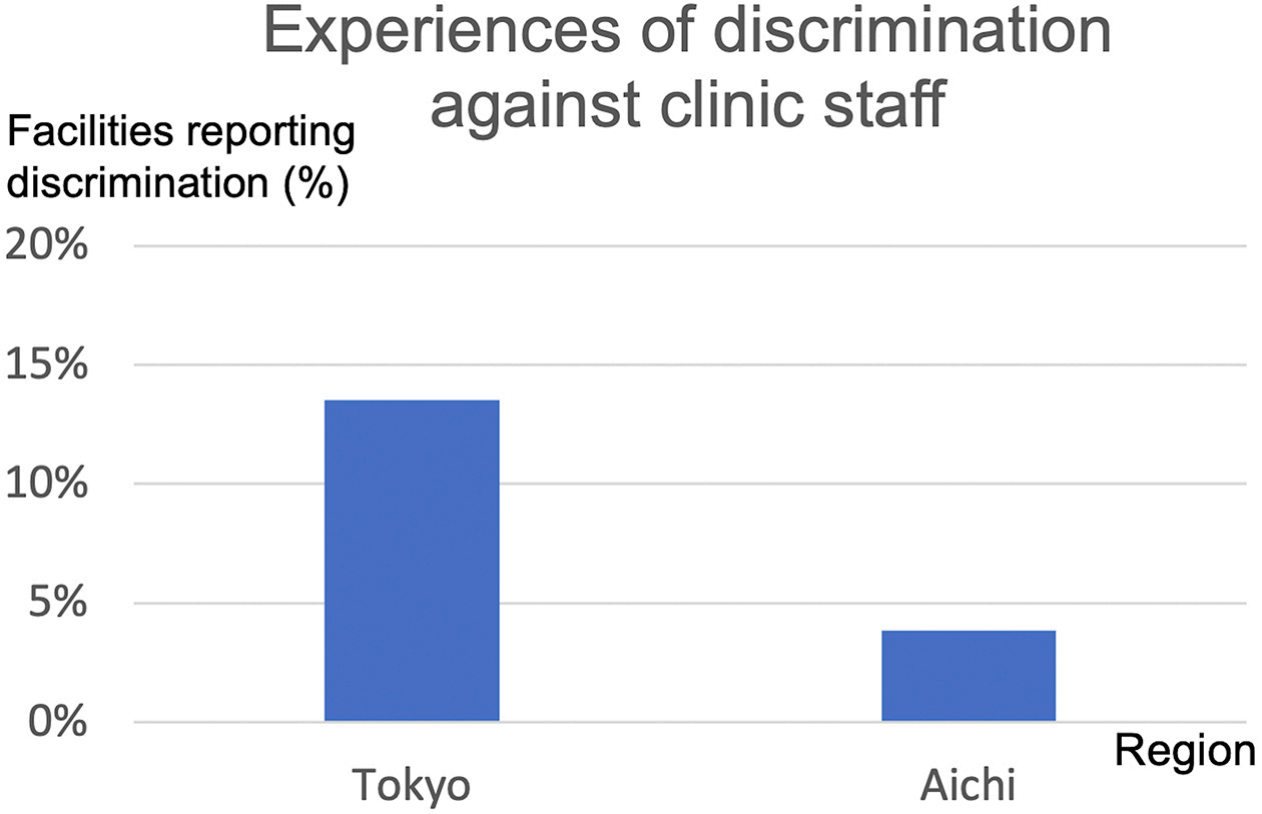
Experiences of discrimination against clinic staff since the COVID-19 pandemic. Bars indicate the proportion of clinics reporting discrimination, stratified by region (Tokyo and Aichi Prefecture). Comparisons between regions were performed using Fisher’s exact test (p = 0.387). COVID-19: coronavirus disease 2019.

A small proportion of clinics reported experiences of discrimination against clinic staff since the COVID-19 pandemic (Figure 3). When stratified by region, the proportion appeared higher in Tokyo than in Aichi Prefecture; however, this difference was not statistically significant (Fisher’s exact test, p = 0.387).

Regarding operational changes following the reclassification of COVID-19 as a Category 5 infectious disease in Japan, facilities reported relaxing several infection control measures, including optional mask use, and discontinuing routine use of face shields or medical gowns. Some facilities also reported changes in testing locations and shortened isolation periods for infected individuals. Additionally, several facilities described financial and operational pressures following the discontinuation of COVID-19-related subsidies.

## Discussion

This multi-facility questionnaire study provides insight into how outpatient clinics in Japan operationally implemented infection control measures for staff during the COVID-19 pandemic. Several measures were rapidly and widely adopted across facilities, suggesting that they represent practical baseline strategies for protecting clinic staff during infectious disease outbreaks.

Healthcare workers experienced a high risk of infection and a significant psychological burden during the pandemic ^(2), (5)^. Our findings indicate that, despite limited resources, outpatient clinics were able to implement infection control measures that enabled continued medical care. With regard to PPE, our findings indicate that a substantial proportion of outpatient clinics implemented measures such as the use of face shields, gloves, and protective clothing. However, the overall provision rate of PPE was not universal, suggesting that some clinics relied on selective or situational use rather than comprehensive implementation. This pattern likely reflects practical constraints in outpatient settings, including limited resources, space, and staffing, particularly during periods of high demand. Importantly, no statistically significant regional differences were observed in the provision of PPE between clinics in Tokyo and Aichi Prefecture. This suggests that basic PPE-related infection control practices were adopted in a broadly similar manner across different outpatient clinic settings, regardless of regional characteristics. These findings highlight that, even under constrained conditions, outpatient clinics were able to maintain a baseline level of infection control while adapting PPE use to local operational needs.

A notable finding of this study was the significantly higher use of digital communication tools for information sharing among clinic staff in Tokyo compared with Aichi Prefecture. This regional difference may be explained by several contextual factors. Clinics in metropolitan areas such as Tokyo often manage larger patient volumes and more complex workflows, which may increase the need for rapid and efficient communication among staff. In such settings, digital tools―including messaging applications, e-mail, and web-based platforms―can facilitate timely dissemination of updates related to infection control policies and operational changes.

In addition, regional differences in information and communication technology infrastructure and staff demographics may have contributed to this finding. Urban clinics may have greater access to digital resources and a higher proportion of staff familiar with digital communication platforms. Furthermore, support and guidance provided by local medical associations may influence the adoption of specific information-sharing practices. Although the present study was not designed to evaluate these factors directly, the observed regional difference suggests that flexible communication strategies tailored to local contexts may enhance preparedness for future infectious disease outbreaks.

Although the overall proportion of clinics reporting discrimination against staff was small, this issue warrants attention. The absence of a statistically significant regional difference suggests that experiences of discrimination may not be confined to specific geographic settings. However, clinics in metropolitan areas may face greater public exposure and patient volume, potentially increasing opportunities for such experiences. Given the limited sample size and low event frequency, these findings should be interpreted with caution. Nevertheless, addressing stigma and discrimination against healthcare workers remains an important consideration in pandemic preparedness and response ^(5), (6)^.

The World Health Organization has highlighted that protecting healthcare workers is essential to sustaining healthcare systems during infectious disease outbreaks ^(6)^. Our findings support the need for outpatient clinics to maintain the capacity to rapidly reintroduce enhanced infection control measures should future emerging infectious diseases occur.

This study has limitations, including reliance on self-reported data and a focus on specific regions in Japan. Nevertheless, it provides valuable real-world evidence from outpatient clinic settings, which have been underrepresented in existing literature.

### Conclusions

In conclusion, outpatient clinics in Japan implemented a range of operational infection control strategies for clinic staff during the COVID-19 pandemic, despite limited physical space and resources. These strategies included standardized baseline measures, such as the use of physical barriers, basic PPE, and staff health monitoring, which were adopted across regions.

At the same time, clinics employed flexible, context-dependent approaches tailored to their local environments. Examples of such strategies included the use of digital communication tools for timely information sharing among staff, and adjustments in operational workflows to accommodate infection control needs under resource constraints. The combination of standardized baseline measures with adaptable strategies may enhance the resilience of outpatient clinics and inform preparedness planning for future emerging infectious diseases.

## Article Information

### Acknowledgments

The authors would like to thank the members of the Minato Ward Medical Association in Tokyo and the Higashi Nagoya Medical Association in Aichi Prefecture for their responses to the survey. I would also like to take this. I would also like to thank the Medical Safety Promotion Organization Higher Education Program of Medical Safety (MSPO-HEPMS) student members for their advice.

### Author Contributions

Kei Ijichi and Hiroshi Yotsuyanagi conceived and designed the study, collected the data, performed the statistical analysis, interpreted the results, and drafted the manuscript. The author approved the final version of the manuscript.

### Conflicts of Interest

None

### Institutional Review Board Approval

This study was approved by the Institutional Review Board of the Institute of Medical Science, University of Tokyo (Approval No. 2023-23-0720).

### Ethical Approval

The Institute of Medical Science, The University of Tokyo’s ethical review board, approved this research. (2023-23-0720).
